# Human pituitary tumor-transforming gene 1 overexpression reinforces oncogene-induced senescence through CXCR2/p21 signaling in breast cancer cells

**DOI:** 10.1186/bcr3226

**Published:** 2012-07-12

**Authors:** Jhen-Wei Ruan, Yi-Chu Liao, Ingrid Lua, Ming-Hsun Li, Chih-Yi Hsu, Ji-Hshiung Chen

**Affiliations:** 1 Institute of Medical Science, Tzu-Chi University, No.701, Sec. 3, Zhongyang Rd., Hualien, 97004, Taiwan; 2 Department of Molecular Biology and Human Genetics, Tzu-Chi University, No.701, Sec. 3, Zhongyang Rd., Hualien, 97004, Taiwan; 3 Department of Pathology, Buddhist Tzu-Chi General Hospital, No.707, Sec. 3, Zhongyang Rd., Hualien, 97002, Taiwan; 4 Department of Pathology and Laboratory Medicine, Taipei Veterans General Hospital, No.201, Sec. 2, Shipai Rd., Taipei, 11217, Taiwan

## Abstract

**Introduction:**

*hPTTG1 *(human pituitary tumor-transforming gene 1) is an oncogene overexpressed in breast cancer and several other types of cancer. Increased hPTTG1 expression has been shown to be associated with poor patient outcomes in breast cancer. Although hPTTG1 overexpression plays important roles in promoting the proliferation, invasion, and metastasis of cancer cells, it also has been suggested to induce cellular senescence. Deciphering the mechanism by which hPTTG1 overexpression induces these contradictory actions in breast cancer cells is critical to our understanding of the role of hPTTG1 in breast cancer development.

**Methods:**

MCF-10A and MCF-7 cells were used to identify the mechanism of hPTTG1-induced senescence. The interplay between hPTTG1 overexpression and chemokine C-X-C motif receptor 2 (CXCR2)/p21-dependent senescence in tumor growth and metastasis of MCF-7 cells was investigated by orthotopic transplantation of severe combined immunodeficiency (SCID) mice. Additionally, human invasive ductal carcinoma (IDC) tissue arrays were used to confirm the hPTTG1/CXCR2/p21 axis established *in vitro*.

**Results:**

In this study, we investigated the mechanism of hPTTG1-induced senescence as well as its role in breast cancer progression and metastasis. Herein, we showed that hPTTG1 overexpression reinforced senescence through the CXCR2/p21 signaling. Furthermore, hPTTG1 overexpression activated NF-κB signaling to transactivate the expression of interleukin (IL)-8 and growth-regulated oncogene alpha (GROα) to execute CXCR2 signaling in MCF-7 cells. When CXCR2 expression was knocked down in hPTTG1-overexpressing MCF-7 cells, hPTTG1-induced senescence was abrogated by alleviating CXCR2-induced p21 expression. In a mouse model, CXCR2-mediated senescence limited hPTTG1-induced tumor growth and metastasis. Moreover, CXCR2 knockdown in hPTTG1-overexpressing MCF-7 tumors dramatically accelerated tumor growth and metastasis. Our *in vitro *and *in vivo *results demonstrated that hPTTG1 overexpression reinforces senescence through CXCR2 signaling, and the evasion of CXCR2/p21-dependent senescence was critical to hPTTG1 exerting its oncogenic potential. Interestingly, although CXCR2-dependent senescence restrained hPTTG1-induced tumor progression, when MCF-7 cells and hPTTG1-overexpressing MCF-7 cells were co-transplanted into the mammary fat pads of SCID mice, hPTTG1-overexpressing senescent cells created a metastasis-promoting microenvironment that promoted lung metastasis of the MCF-7 cells. Immunohistochemical analysis of human breast tumor samples also confirmed the importance of the hPTTG1/CXCR2 axis in promoting breast cancer metastasis.

**Conclusions:**

Our findings provide novel molecular insights into hPTTG1-induced senescence and identify a novel mechanism by which hPTTG1 promotes metastasis by regulating the senescence-associated microenvironment.

## Introduction

*PTTG1 *is an oncogene first isolated from rat pituitary tumor cells [1]. In mouse 3T3 fibroblasts, PTTG1 overexpression limits cell proliferation but promotes cell transformation [1]. In human cells, hPTTG1 has been characterized as a cell-cycle regulator that regulates the separation of sister chromatids [2] and plays important roles in cancer cell transformation and tumorigenesis [3]. Increased expression of hPTTG1 has been observed to be associated with poor prognosis in several types of human cancers [4-8]. In breast cancer, hPTTG1 overexpression is a proliferation marker [9] and can be considered a prognostic marker or a molecular signature of metastasis and recurrence [10,11].

When hPTTG1 is overexpressed, it exerts an oncogenic potential to enhance the proliferation, invasion, and metastasis of cancer cells [12-15]. In our previous study, we demonstrated that hPTTG1 can regulate the actin cytoskeletal dynamics of breast cancer cells by activating GEF-H1/RhoA signaling to promote breast cancer metastasis [13]. In contrast to its oncogenic properties, hPTTG1 also is suggested to be involved in the regulation of senescence. In pituitary tumor cells, either the depletion or overexpression of hPTTG1 induces chromosomal instability and executes p21-dependent senescence [16-18]. In human fibroblasts and colorectal cancer cells, hPTTG1 overexpression induces the DNA-damage response (DDR) to trigger p53-dependent senescence [19]. Deciphering the mechanism by which hPTTG1 overexpression regulates these contradictory actions is critical to our understanding of the role of hPTTG1 in cancer development.

Oncogene-induced senescence (OIS) is a barrier to oncogene-induced tumor growth and malignant progression that works by placing cancer cells into an irreversible state of cell-cycle arrest [20,21]. Although senescence has dramatic tumor-suppression effects via the inhibition of tumor growth, accumulating evidence suggests that senescence may be both beneficial and detrimental for patient outcomes [20,22,23]. To reinforce OIS, cells secrete a number of cytokines and chemokines to construct a senescence-promoting microenvironment [24,25]; this secretory behavior is known as the senescence-associated secretory phenotype (SASP). For example, senescent human fibroblasts increase their expression of IL-8 and GROα to activate CXCR2 signaling, which reinforces senescence [25]. However, the misuse of SASP factors by cancer cells may support their proliferation and induce a chronic inflammatory response [26].

CXCR2 is a G protein-coupled receptor that is activated by CXC chemokines including IL-8, GROα, GROβ, and GROγ. The increased expression of CXCR2 has been suggested to promote tumor growth, angiogenesis, and metastasis in head and neck cancer, non-small-cell lung cancer, ovarian cancer, and melanoma [27]. IL-8/CXCR2 signaling exerts its oncogenic potential by activating the Akt/mTOR, MAPK, and androgen-receptor pathways [28]. Similar to hPTTG1, CXCR2 promotes malignant progression in certain types of cancer and triggers senescence in human normal fibroblasts [25]. These findings suggest that CXCR2 plays important roles in both tumor suppression and promotion. In breast cancer, the role of CXCR2 in cancer development and metastasis remains unclear [29].

In this study, we aimed to elucidate the mechanism of hPTTG1-induced senescence and to understand its role in breast cancer progression. Herein, we provided a novel link between hPTTG1 overexpression and CXCR2-dependent senescence. In breast cancer cells, hPTTG1 overexpression reinforces OIS through a CXCR2/p21 signaling. Although CXCR2-dependent senescence dramatically inhibits the tumor growth of hPTTG1-overexpressing MCF-7 cells, hPTTG1-induced SASP remodels the tumor microenvironment to promote the metastasis of neighboring nonsenescent cancer cells. In summary, we provide novel insights into how hPTTG1-induced senescence contributes to breast cancer metastasis.

## Materials and methods

### Cell culture and reagents

The following cell lines were used in this study: breast cancer cell lines MDA-MB-231, MCF-7, T-47D, and AU 565; human normal mammary epithelial cell line MCF-10A; and colorectal cancer cell lines HCT116 and *p53-*null HCT116. MDA-MB-231, MCF-7, T-47D, and AU 565 cells were grown in Dulbecco modified Eagle medium (DMEM) (Sigma-Aldrich, St. Louis, MO, USA) containing 10% fetal bovine serum. MCF-10A cells were grown in DMEM/F12 medium (GIBCO BRL, Eggenstein, Germany) containing 5% horse serum, 10 μg/ml insulin, 20 ng/ml EGF, 100 ng/ml cholera toxin, 0.5 μg/ml hydrocortisone, and 100 units/ml penicillin/streptomycin. HCT116 and *p53*-null HCT116 colorectal tumour cells (from Dr. Bert Vogelstein of the Johns Hopkins School of Medicine) were maintained in McCoy 5A medium containing 10% fetal bovine serum. Cells were grown at 37°C in a 5% CO_2 _atmosphere. The *hPTTG1 *expression plasmid was obtained from our laboratory [13]. The *CXCR2 *expression plasmid was purchased from ORIGENE Inc. (SC323957; Rockville, MD, USA) and subcloned into the pcDNA3.1 vector (Invitrogen, Carlsbad, CA, USA).

SB225002 (an antagonist of CXCR2, 10 μ*M*; Calbiochem, San Diego, CA, USA) or JSH-23 (an inhibitor of the NF-κB transcription factor, 10 μ*M*; Calbiochem) was added to the cells, and 0.001% DMSO (vehicle) was added as a control. For neutralization of CXCR2 and IL-8, anti-CXCR2 antibody (5 μg/ml, MAB331; R&D Systems Inc., Minneapolis, MN, USA) or anti-IL-8 antibody (1 μg/ml, AF-208-NA; R&D Systems) was added to the cells, and isotype anti-IgG antibody (5 μg/ml, MAB003; R&D Systems) was added as a control (Control IgG).

### Generation of stable selected cells

To generate the stable selected pools, MCF-7 and MCF-10A cells were stably transfected with 4 μg of the pcDNA3.1 empty vector (stable mock-transfected MCF-7/MCF-10A cells) or the pcDNA3.1-*hPTTG1 *expression plasmid (stable hPTTG1-overexpressing MCF-7/MCF-10A cells) by using Lipofectamine 2000 (Invitrogen) and were then selected with hygromycin (100 μg/ml).

For the puromycin selection of stable knockdown cells, lentiviruses encoding specific shRNAs were produced by following the established protocol provided by the National RNAi Core Facility, Academia Sinica (Taipei, Taiwan) [30]. The specific shRNA reagents targeting p53 and p21 (shp53-1:TI379448, shp53-2:TI379451, shp21-1:TI321870, and shp21-2:TI321871) were purchased from Origene Inc. (Rockville, MD, USA). The specific shRNA reagents targeting CXCR2 (shCXCR2-1:TRCN0000009138, shCXCR2-2:TRCN0000009136) and GFP (shGFP: TRCN0000072178) were obtained from the National RNAi Core Facility, Academia Sinica (Taipei, Taiwan).

### Immunoblot analysis

The immunoblot analysis was performed as previously described [13] with antibodies against Rb (no. 9309), p-Rb (9307), p21 (2947), IKKα (2682), IKKβ (2370), p-p65 (3033), p65 (8242), p-Erk1/2 (4370), p-p53^ser15 ^(9286), γ-H2AX (9718) (Cell Signaling Technology, Beverly, MA, USA), p53 (SC-126), p16^INK4A ^(SC-1661), Erk1/2 (SC-94), β-actin (SC-1616) (Santa Cruz Biotechnology, Santa Cruz, CA, USA), or hPTTG1 (34-1500, Invitrogen).

### Cytokine array

Cytokine arrays were used to determine the relative levels of 36 different cytokines and chemokines. The array was performed by using the protocol provided by the manufacturer (Ary005; R&D Systems). After development, the films were scanned, and the images were quantified by using Image J (National Institutes of Health, USA).

### Senescence-associated β-galactosidase (SA-β-Gal) assay

SA-β-Gal assays were performed according to the manufacturer's instructions (Millipore, Billerica, MA, USA). In brief, the cells were fixed and incubated at 37°C in a solution containing 1 mg/ml 5-bromo-4-chloro-3-indolyl-b-D-galactoside (X-gal) for 12 hours. After staining, 250 cells were counted for each sample.

### Luciferase reporter assays

The 5' regulatory sequences of CXCR2 (nucleotides -1,469 to +40, which spans 1,469 bp upstream to 40 bp downstream of the transcription start site), IL-8 (nucleotides -875 to +1, which spans 875 bp upstream to 1 bp downstream of the transcription start site), and GROα (nucleotides -1,442 to +52, which spans 1,442 bp upstream to 52 bp downstream of the transcription start site) were polymerase chain reaction (PCR) amplified from human genomic DNA and inserted into the pGL4 luciferase vector (Promega, Madison, WI, USA). The IL-8 regulatory sequence (pGL4-IL8) contains one NF-κB binding site (-82/-72). In the mutated IL-8 promoter (pGL4-IL8M), the NF-κB binding sequence was replaced with the Bgl II sequence (AGATCT) with a standard cloning procedure. The GROα regulatory sequence (pGL4-GROα) contains one NF-κB binding site (-77/-67). The NF-κB binding site deleted mutant (pGL4-GROαM) spans 1,442 bp upstream to 80 bp upstream of the transcription start site of the GROα regulatory sequence (-1,442/-80). MCF-7 cells were transfected by using Lipofectamine 2000 (Invitrogen), and the luciferase activities were measured 48 hours after transfection by using the Dual-Luciferase Reporter System (Promega).

### RNA isolation and quantitative RT-PCR (qRT-PCR)

Cytosolic RNA was isolated from confluent cultures in 10-cm cell cultures with a Trizol reagent kit (Invitrogen) according to the manufacturer's instructions. RNA was converted to cDNA with the SuperScript III First-Strand Synthesis System (Invitrogen). qRT-PCR was performed according to the manufacturer's instructions by using ABI 7300 (Applied Biosystems).

### FACS (fluorescence-activated cell sorting) analysis

The expression of CXCR2 was evaluated in cells obtained from cell culture with a FACScan (Becton Dickinson, San Jose, CA, USA). A fluorescein isothiocyanate (FITC)-conjugated anti-CXCR2 antibody (no. 551126) or FITC-conjugated isotype anti-IgG antibody (no. 555748) (Becton Dickinson) was used in the staining procedure according to the instructions of the manufacturer.

### ELISA (enzyme-linked immunosorbent assay)

Conditioned media were collected from 5 × 10^5 ^cells after a 48-hour incubation. The concentrations of IL-8 and GROα in the supernatant were measured with specific ELISA kits (PEPROTECH, Rocky Hill, NJ, USA)

### BrdU and cell-growth assays

BrdU colorimetric assays were performed by following the manufacturer's instructions (Roche, Mannheim, Germany). In brief, cells were labeled with BrdU for 8 hours. After labeling, the cells were fixed and the DNA denatured. A monoclonal anti-BrdU antibody was used to bind the incorporated BrdU. After washing, TMB substrates were added, and the absorbance measured in an ELISA reader at 370 nm. For the cell-growth assay, 5 × 10^4 ^cells were seeded in six-well plates, and growth curves were obtained by daily cell counts.

### ChIP assay

The ChIP assay was performed according to the protocol of ChIP assay kit (Millipore). The chromatin preparation and ChIP reactions were performed as previously described [13]. qRT-PCR was used to enrich promoter binding levels, and those data are expressed as fold enrichment (fold increase over the control IgG). qRT-PCR was carried out with the following primers: IL-8 p65 binding site forward, 5'-GGTTTGCCCTGAGGGGATG-3' and reverse, 5'-CCTACTAGAGAACTTATGCACCCTCATC-3'; GROα p65 binding site forward, 5'- GGACTCGGGATCGATCTGG-3' and reverse, 5'-GTGGCTCTCCGAGATCCGC-3'.

### Animal studies

All experiments involving mice were performed with approval of the Laboratory Animal Center at Tzu-Chi University. For the spontaneous metastasis assay, 5 × 10^5 ^cells were injected into the fourth mammary fat pad of 5- to 8-week-old SCID mice in 1:1 Matrigel (Becton Dickinson) plus PBS. Tumor growth on orthotopic implantation was measured weekly. Lungs from mice bearing the indicated tumors were isolated after 12 and 14 weeks. All dissected lungs were paraffin-embedded, sectioned, and stained with H&E, and the metastatic nodules were counted as previously described [13].

### Immunohistochemistry

Tissue samples from patients with breast cancer (100 patients, Biomax, BC081120) and those with breast cancer with matched metastatic carcinoma of the lymph node (50 patients, Biomax, BR1005) were purchased from US Biomax, Inc. (Rockwell, MD, USA). All tissues were collected by Biomax according to the HIPAA-approved protocol for ethical standards after receiving patient consent. IHC was performed as previously described [13]. The appropriate volume of diluted primary anti-hPTTG1 (1:100, 34-1500, Invitrogen), anti-CXCR2 (1:80, ab14935, Abcam) or anti-p21 (1:50, 2947, Cell Signaling) antibody was added to cover the specimen, and the samples were incubated at 4°C overnight. Nuclei were then counterstained with hematoxylin.

To evaluate the expression levels of hPTTG1, CXCR2, and p21, immunostained human breast tissues or metastatic carcinomas were judged by two pathologists by using the following criteria. The criteria for hPTTG1 evaluation were described in our previous study [13]. For p21, the classifications included 3^+ ^(strong p21 nuclear staining in > 10% tumor cells), 2^+ ^(positive p21 nuclear staining in > 5% tumor cells), and 0/1^+ ^(no or positive p21 nuclear staining in < 5% tumor cells). CXCR2 was classified as strong (3^+^), moderate (2^+^), or weak (0/1^+^) based on the intensity of both membrane and cytoplasmic immunoreactivity. The association of the expression of hPTTG1 with CXCR2 expression in invasive breast carcinoma was analyzed by using the Pearson χ^2 ^correlation test.

### Migration and invasion assays

For the migration assays, stable ZsGreen-labeled MCF-7 cells (0.5 × 10^5^) and stable mock-transfected MCF-7 (0.5 × 10^5^) or stable hPTTG1-overexpressing MCF-7 (0.5 × 10^5^) cells were mixed and seeded in a uncoated membranes with 8.0-μm pores (Corning, Santa Clara, CA, USA). For the invasion assays, stable ZsGreen-labeled MCF-7 cells (0.5 × 10^5^) and stable mock-transfected MCF-7 (0.5 × 10^5^) or stable hPTTG1-overexpressing MCF-7 (0.5 × 10^5^) cells were mixed and seeded in a Matrigel-coated chamber with 8.0-μm pores (Becton Dickinson). Cells were seeded in serum-free medium and migrated toward the growth media containing 1% fetal bovine serum for 48 hours. The fluorescent migrating or invading cells in polycarbonate membranes were counted, as previous reported [31].

### Statistical analysis

Data are presented as the mean ± SEM unless otherwise noted. The Student *t *test was used for comparisons. A level of *P *< 0.05 was considered significant.

## Results

### hPTTG1 overexpression reinforces senescence in breast cancer and normal breast epithelial cells

**Figure 1:**
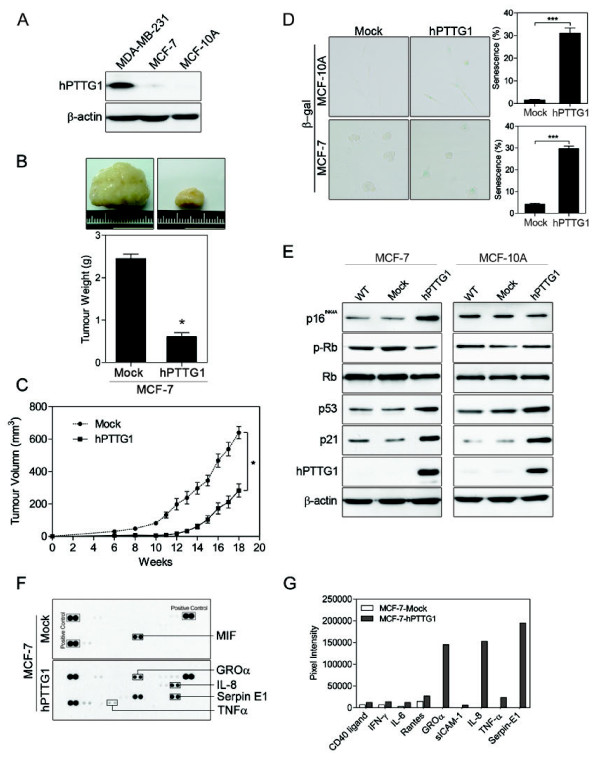
**hPTTG1 overexpression reinforces senescence and senescence-associated secretory phenotype (SASP)**. **(A) **Cell lysates of MDA-MB-231, MCF-7, and MCF-10A cells were collected for immunoblot analysis. **(B) **MCF-7 cells stably transfected with pcDNA3.1 (Mock; MCF-7^Mock^) or pcDNA3.1-*hPTTG1 *(hPTTG1; MCF-7^hPTTG1^) were orthotopically injected into fourth mammary fat pads of SCID mice. The primary tumor weights were measured 18 weeks after implantations. Representative tumors are shown in the upper panel. Quantitative results are shown in the lower panel (*n *= 6 per group). The results are presented as the mean ± SEM. *P < 0.05. **(C) **Tumor growth was measured weekly after orthotopic implantations of MCF-7 cells stably transfected with pcDNA3.1 (Mock) or pcDNA3.1-*hPTTG1 *(hPTTG1). The experiments were terminated 18 weeks after implantation. Representative tumors are shown in (B). *n *= 6 per group per time point. Each time point shows the mean ± SEM. *P < 0.05. **(D) **MCF-7 and MCF-10A cells stably transfected with pcDNA3.1 (Mock) or pcDNA3.1-*hPTTG1 *(hPTTG1) were plated for the senescence assay. The percentage of positively stained cells is indicated to the right of the respective images. Data represent mean ± SEM (*n *= 3). ***P < 0.0001. **(E) **Cell lysates were collected from MCF-7 and MCF-10A cells stably transfected with pcDNA3.1 (Mock) or pcDNA3.1-*hPTTG1 *(hPTTG1) for immunoblot analysis. WT, untransfected cells. **(F) **Conditioned media were collected from MCF-7 cells stably transfected with pcDNA3.1 vector (Mock) or pcDNA3.1-*hPTTG1 *(hPTTG1) for cytokine array analysis. The differences are indicated with boxes. **(G) **The pixel intensity of (F) was analyzed by using Image J software.

In our previous study, we found hPTTG1 to be endogenously overexpressed in the metastatic MDA-MB-231 breast cancer cell line and expressed at very low levels in the nonmetastatic MCF-7 breast cancer cell line and normal mammary epithelial cells [13] (Figure 1A). In addition, hPTTG1 overexpression enhanced the motility and invasiveness of both MDA-MB-231 and MCF-7 cells by activating GEF-H1/RhoA signaling [13]. Interestingly, although knockdown of hPTTG1 expression in MDA-MB-231 cells attenuated the tumor growth of these cells in an orthotopic mouse model [13], ectopic overexpression of hPTTG1 in MCF-7 cells also significantly reduced the tumor size and growth rate of MCF-7 cells (Figure 1B, C).

Because hPTTG1 overexpression has been demonstrated either to inhibit cell proliferation [1,32] or to induce senescence [17,19], we therefore asked whether hPTTG1 overexpression induces senescence in breast cancer cells. To assess the effect of hPTTG1 overexpression on senescence in breast cancer, we used MCF-10A normal breast epithelial cells and MCF-7 breast cancer cells, both of which endogenously express very low levels of hPTTG1 (Figure 1A). By measuring the activity of senescence-associated β-galactosidase (SA-β-Gal), we found that hPTTG1 overexpression significantly reinforced senescence in both cell lines (Figure 1D). hPTTG1 overexpression has been shown to induce senescence through p53- or p21-dependent pathways [17,19]. Indeed, in both cells, hPTTG1 overexpression increased the expression of the p53 and p21 (Figure 1E). In addition, hPTTG1 overexpression increased the expression of the p16^INK4A ^in MCF-7 cells but not in MCF-10A cells, and the expression and phosphorylation of Rb was not significantly altered by hPTTG1 overexpression in both cell lines (Figure 1E). p53/p21 and p16/pRb are two major pathways involved in OIS regulation [21]. These results suggest that hPTTG1 overexpression may reinforce senescence through p53/p21 but not p16/pRb signaling in breast cancer and normal breast epithelial cells.

SASP is a specific characteristic of senescent cells responsible for generating the senescence-promoting microenvironment [26]. To confirm further whether hPTTG1 overexpression can induce SASP in breast cancer cells, we used antibody arrays to monitor the expression profile of secreted cytokines in the conditioned media from stable mock-transfected (MCF-7^Mock^) and stable hPTTG1-overexpressing MCF-7 cells (MCF-7^hPTTG1^, MCF-7 cells stably transfected with pcDNA3.1-*hPTTG1 *expression plasmid). As expected, hPTTG1 overexpression specifically induced the expression of certain inflammatory cytokines and chemokines, including IL-8, GROα, TNF-α, and Serpin E1 (Figure 1F, G). These results indicate that hPTTG1 overexpression induces senescence and SASP in breast cancer cells.

### hPTTG1 overexpression co-upregulates the CXCR2 receptor and its ligands

**Figure 2:**
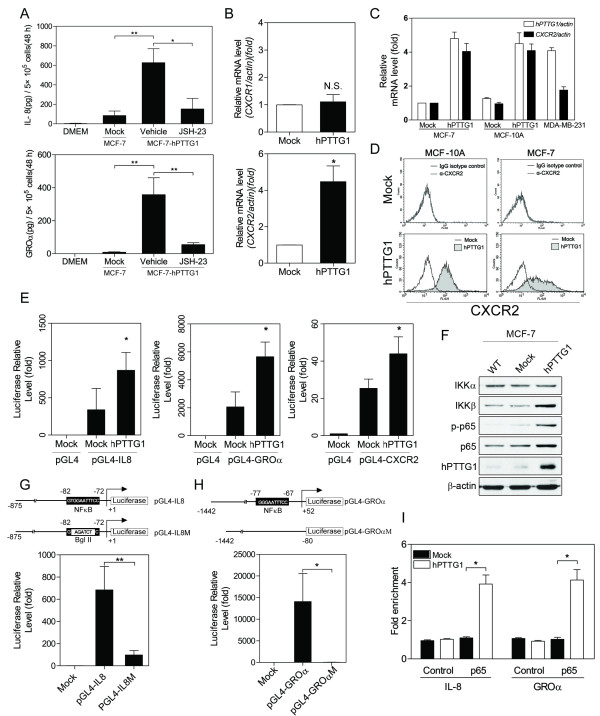
**hPTTG1 overexpression upregulates the expression of CXCR2, IL-8, and GROα**. **(A) **Conditioned media were collected from MCF-7^Mock ^or MCF-7^hPTTG1 ^cells treated with vehicle control (DMSO) or an NF-κB inhibitor (JSH-23) for ELISA analysis of IL-8 (upper panel) and GROα (lower panel). Unconditioned DMEM media acted as negative control. **(B) ***CXCR1 *(upper panel) *and CXCR2 *(lower panel) mRNA expression was analyzed with qRT-PCR in MCF-7^Mock ^and MCF-7^hPTTG1 ^cells. **(C) ***hPTTG1 *and *CXCR2 *mRNA expression was analyzed with qRT-PCR in MCF-7^Mock^, MCF-7^hPTTG1^, MCF-10A^Mock^, MCF-10A^hPTTG1^, and MDA-MB-231 cells. **(D) **MCF-10A^Mock^, MCF-10A^hPTTG1^, MCF-7^Mock^, and MCF-7^hPTTG1 ^cells were stained with a FITC-conjugated anti-CXCR2 antibody for FACS analysis. **(E) **The regulatory sequences of IL-8, GROα, and CXCR2 were co-transfected with pcDNA3.1 (Mock) or pcDNA3.1-*hPTTG1 *(hPTTG1) into MCF-7 cells for luciferase reporter assays. **(F) **Cell lysates were collected from untransfected MCF-7 (WT), MCF-7^Mock^, and MCF-7^hPTTG1^cells for immunoblot analysis. **(G) **The pGL4 vector (Mock), IL-8 promoter, or the mutated IL-8 promoter was transiently cotransfected with pcDNA3.1-*hPTTG1 *expression plasmid into MCF-7 cells for luciferase reporter assays. **(H) **The pGL4 vector (Mock), GROα promoter, or the NF-κB binding site deleted mutant was transiently cotransfected with the pcDNA3.1-*hPTTG1 *expression plasmid into MCF-7 cells for luciferase reporter assays. **(I) **ChIP assays were performed on MCF-7^Mock ^or MCF-7^hPTTG1 ^cells. The ChIP-qPCR data are expressed as the fold increase over the control (IgG) for the promoter region containing p65-binding elements. The results in A to C, E, and G to I are presented as the mean ± SEM (*n *= 3). *P < 0.05; **P < 0.01; NS, not significant.

With antibody arrays, we determined that hPTTG1 overexpression led to a considerable induction in IL-8 and GROα. Because the antibody arrays were semiquantitative, we performed ELISA (enzyme-linked immunosorbent assay) analyses to confirm this result (Figure 2A). Both IL-8 and GROα are ligands of CXCR1/2 receptors, and the IL-8/CXCR2 axis has been reported to reinforce OIS [25]. Thus, we next investigated whether hPTTG1 could upregulate the expression of CXCR2 during hPTTG1-induced senescence. By using quantitative RT-PCR (qRT-PCR), we found that hPTTG1 overexpression significantly induced the mRNA expression of CXCR2 but not CXCR1 in MCF-7 cells (Figure 2B). By using qRT-PCR and fluorescence-activated cell sorting (FACS) analysis, we confirmed that the expression level of CXCR2 was significantly induced by hPTTG1 overexpression in both MCF-10A and MCF-7 cells that endogenously express both hPTTG1 and CXCR2 at very low levels (Figure 2C, D).

To investigate further the mechanism by which hPTTG1 upregulates the expression of CXCR2 and its ligands, we performed luciferase reporter analysis on 5' upstream regulatory sequences of CXCR2, IL-8, and GROα. The results demonstrated that hPTTG1 overexpression enhances the transcriptional activities of the CXCR2, IL-8, and GROα promoters (Figure 2E), suggesting that hPTTG1 co-upregulates the expression of CXCR2 and its ligands through transcriptional regulation.

NF-κB is a core transcription-factor complex involved in the inflammatory response and cancer development [33]. The activation of NF-κB signaling plays important roles in enhancing SASP when senescence is induced [23,34]. To elucidate further how hPTTG1 regulates the expression of CXCR2 ligands, we screened the expression and phosphorylation status of proteins associated with the NF-κB pathway. The results demonstrated that hPTTG1 overexpression increased the expression of IKKβ but not IKKα (Figure 2F). Moreover, hPTTG1 overexpression enhanced the phosphorylation of p65, which is the major component of the NF-κB complex (Figure 2F). This result supports the previous finding that IKKβ and p65 play roles in the regulation of CXCR2 ligands [25]. Furthermore, in hPTTG1-overexpressing MCF-7 cells, deletions of the NF-κB binding site [35,36] eliminated the luciferase activity of the IL-8 and GROα promoters (Figure 2G, H). These results were further confirmed by ChIP (chromatin immunoprecipitation) assay, which revealed that the binding capacities of p65 were enhanced by hPTTG1 overexpression (Figure 2I). In addition, when MCF-7^hPTTG1 ^cells were treated with a p65-specific inhibitor (JSH-23), the secretion of IL-8 and GROα was also alleviated (Figure 2A). These results indicate that hPTTG1 overexpression activates NF-κB signaling to enhance the expression of IL-8 and GROα.

### hPTTG1-induced senescence is CXCR2/p21 dependent

**Figure 3:**
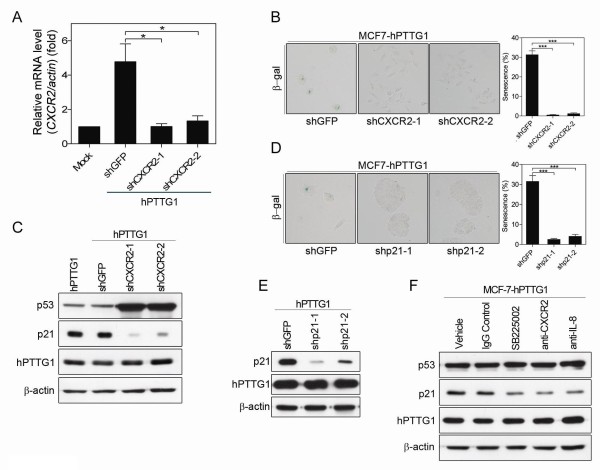
**hPTTG1 overexpression activates CXCR2/p21 signaling to induce senescence**. **(A) **The level of CXCR2 knockdown was confirmed with qRT-PCR. The results are presented as the mean ± SEM (*n *= 3). *P < 0.05. **(B) **MCF-7^hPTTG1 ^cells stably infected with shGFP, shCXCR2-1, or shCXCR2-2 were plated for senescence assays. The percentage of positively stained cells is indicated to the right of the respective images. The results are presented as the mean ± SEM (*n *= 3). ***P < 0.0001. **(C) **Cell lysates were collected from MCF-7^hPTTG1 ^cells and MCF-7^hPTTG1 ^cells stably infected with shGFP, shCXCR2-1, or shCXCR2-2 for immunoblot analysis. **(D) **MCF-7^hPTTG1 ^cells stably infected with shGFP, shp21-1, or shp21-2 were plated for the senescence assay. The percentage of positively stained cells is indicated to the right of the respective images. The results are presented as the mean ± SEM (*n *= 3). ***P < 0.0001. **(E) **Cell lysates were collected from MCF-7^hPTTG1 ^cells stably infected with shGFP, shp21-1, or shp21-2 for immunoblot analysis. **(F) **Cell lysates were collected from MCF-7^hPTTG1 ^cells treated with DMSO (vehicle), IgG control, CXCR2 inhibitor (SB225002), anti-CXCR2 antibody, or anti-IL-8 antibody for immunoblot analysis.

Because hPTTG1 overexpression co-upregulates the expression of CXCR2 and its ligands, we next asked whether hPTTG1 reinforces OIS through CXCR2 signaling. To this end, we knocked down the expression of CXCR2 in MCF-7^hPTTG1 ^cells by using CXCR2-specific shRNA; the level of CXCR2 knockdown was confirmed by qRT-PCR (Figure 3A). Indeed, CXCR2 knockdown dramatically limited the hPTTG1-induced senescence (Figure 3B). CXCR2 reinforces OIS through a p53/p21-dependent pathway [25], and p21 has been indicated to inhibit cell proliferation when hPTTG1 is overexpressed [17,32]. As expected, the depletion of CXCR2 reduced the expression of the p21 protein induced by hPTTG1 overexpression (Figure 3C). Moreover, the depletion of p21 actually abrogated the hPTTG1-induced senescence in MCF-7 cells (Figure 3D, E). When MCF-7^hPTTG1 ^cells were treated with a CXCR2 antagonist (SB225002), or when CXCR2 and IL-8 were neutralized by specific antibodies, the inhibition of IL-8/CXCR2 signaling significantly reduced the hPTTG1-induced p21 expression (Figure 3F). These results demonstrate that hPTTG1 overexpression reinforces senescence through a CXCR2/p21-dependent mechanism.

### The hPTTG1/CXCR2 axis induces p21 expression through a p53-independent mechanism

**Figure 4:**
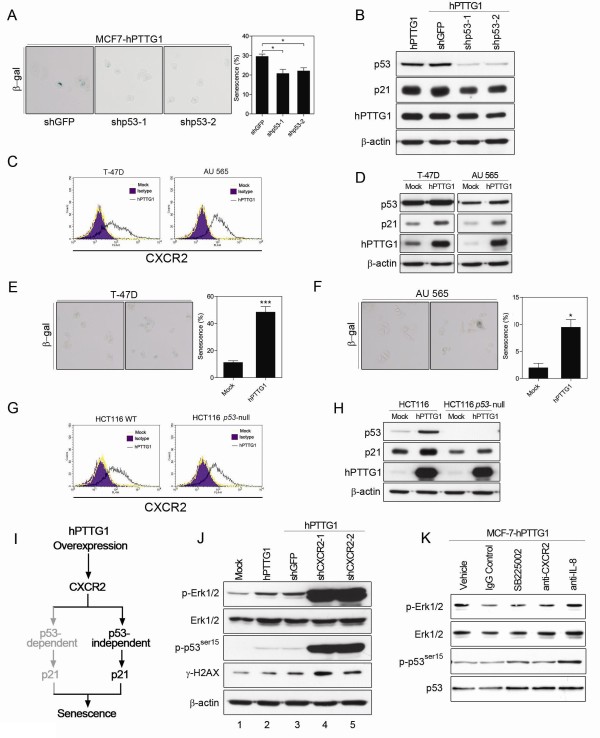
**hPTTG1 overexpression activates CXCR2/p21 signaling through a p53-independent mechanism**. **(A) **MCF-7^hPTTG1 ^cells stably infected with shGFP, shp53-1, or shp53-2 were plated for senescence assays. The percentage of positively stained cells is indicated to the right of the respective images. The results are presented as the mean ± SEM (*n *= 3). *P < 0.05. **(B) **Cell lysates were collected from MCF-7^hPTTG1 ^cells and MCF-7^hPTTG1 ^cells stably infected with shGFP, shp53-1, or shp53-2 for immunoblot analysis. **(C, G) **T47-D, AU 565, HCT116, and *p53-*null HCT116 cells were transiently transfected with pcDNA3.1 (Mock) or pcDNA3.1-*hPTTG1 *(hPTTG1), and then these cells were stained with FITC-conjugated anti-CXCR2 antibody for FACS analysis. **(D, H) **The cell lysates were collected from T47-D, AU 565, HCT116, and *p53-*null HCT116 cells transiently transfected with pcDNA3.1 (Mock) or pcDNA3.1-*hPTTG1 *(hPTTG1) for immunoblot assays. **(E, F) **T47-D and AU 565 cells were transiently transfected with pcDNA3.1 (Mock) or pcDNA3.1-*hPTTG1 *(hPTTG1), and then these cells were plated for senescence assays. The percentage of positively stained cells is indicated to the right of the respective images. The results are presented as the mean ± SEM (*n *= 3). *P < 0.05; ***P < 0.0001. **(I) **Schematic representation of the role of p53 in hPTTG1/CXCR2-mediated senescence. **(J) **Cell lysates were collected from MCF-7^hPTTG1 ^cells and MCF-7^hPTTG1 ^cells stably infected with shGFP, shCXCR2-1, or shCXCR2-2 for immunoblot analysis. **(K) **Cell lysates were collected from MCF-7^hPTTG1 ^cells treated with DMSO (vehicle), IgG control, CXCR2 inhibitor (SB225002), anti-CXCR2 antibody, or anti-IL-8 antibody for immunoblot analysis.

Surprisingly, although *p21 *is a well-known p53-activated gene, CXCR2 depletion reduced the expression of p21 in MCF-7^hPTTG1 ^cells, but p53 expression was not diminished (Figure 3C). To address this issue, we knocked down the expression of p53 in MCF-7^hPTTG1 ^cells. In contrast to CXCR2 depletion, p53 depletion only slightly alleviated hPTTG1-induced senescence (Figure 4A) and hPTTG1-induced p21 expression (Figure 4B). This result suggests that p53 plays only a partial role in hPTTG1/CXCR2-induced senescence and that the hPTTG1/CXCR2 axis may induce p21 expression through a p53-independent mechanism.

hPTTG1 overexpression has been shown to induce p21 expression through a p53-independent mechanism [32]. Therefore, we overexpressed hPTTG1 in T-47D and AU 565 breast cancer cell lines, which carry mutated *p53 *genes [37]. In both T-47D and AU 565 cells, hPTTG1 overexpression induced the expression of CXCR2 and p21 (Figure 4C, D) and significantly reinforced senescence (Figure 4E, F). Furthermore, we also overexpressed hPTTG1 in HCT116 and *p53-*null HCT116 colorectal cancer cell lines. The results demonstrated that hPTTG1 overexpression could induce the expression of CXCR2 and p21 in *p53-*null HCT116 cells (Figure 4G, H). Taken together, these results suggest that hPTTG1 overexpression reinforces CXCR2/21-dependent senescence mainly through a p53-independent mechanism and that the p53-dependent mechanism only minimally affects hPTTG1-induced senescence (Figure 4I).

The results described demonstrated that hPTTG1 overexpression reinforces CXCR2/p21-dependent senescence, but it remains unclear why CXCR2 depletion increases p53 expression in MCF-7^hPTTG1 ^cells. OIS has been recognized as a DDR induced by DNA replication stress [38,39], and both hPTTG1 and CXCR2 are involved in DDRs [19,25]. Therefore, we hypothesized that hPTTG1 overexpression may provoke DDRs to execute senescence. When the CXCR2-dependent senescence pathway is interrupted and fails to arrest the cell cycle, hPTTG1 overexpression may induce higher DNA-replication stress and provoke a greater p53-dependent DDR. We confirmed this hypothesis by monitoring the phosphorylation of H2AX^ser139 ^(γH2AX) and p53^ser15^, which are important phosphorylation signals in DDR. Indeed, hPTTG1 overexpression induced the phosphorylation of γH2AX and p53^ser15 ^in MCF-7 cells (Figure 4J, lane 2). When CXCR2 was depleted, hPTTG1 overexpression dramatically induced stronger phosphorylation of γH2AX and p53^ser15 ^in CXCR2-depleted MCF-7^hPTTG1 ^cells than in MCF-7^hPTTG1 ^cells (Figure 4J, lanes 4 and 5). Notably, depleting CXCR2 in MCF-7^hPTTG1 ^cells also strongly activated the phosphorylation of Erk1/2, a signaling protein closely linked to the proliferation status of cancer cells (Figure 4J). These data indicate that hPTTG1 overexpression induces higher replication stress and provokes a stronger DDR when CXCR2 is depleted. Additionally, the phenomenon that CXCR2 depletion increases p53 expression and alleviates p21 expression in MCF-7^hPTTG1 ^cells provides a possible explanation for why breast cancers with poor prognosis demonstrate increased p53 expression but a loss of p21 expression [40].

Interestingly, unlike the results of CXCR2-depleted MCF-7^hPTTG1 ^cells (Figure 4J), when MCF-7^hPTTG1 ^cells were treated with a CXCR2 antagonist (SB225002) or when CXCR2 and IL-8 were neutralized by specific antibodies, the inhibition of IL-8/CXCR2 signaling did not significantly affect p53 expression or the phosphorylation of ERK1/2 and p53^ser15 ^(Figure 4K). This result may indicate that the CXCR2 antagonist and IL-8/CXCR2 antibodies have a lower inhibitory effect on p21 expression (Figure 3F) as compared with CXCR2 depletion (Figure 3C) and only temporarily inhibit signalling. In this situation, the uninhibited IL-8/CXCR2 signal can still induce a minor senescence response to restrain a portion of the hPTTG1-induced replication stress that provoked DDR. Hence, unlike CXCR2 depletion, the CXCR2 antagonist and IL-8/CXCR2 antibodies did not induce severe DDR in MCF-7^hPTTG1 ^cells.

### CXCR2 modulates the role of hPTTG1 overexpression in tumor growth and metastasis

**Figure 5:**
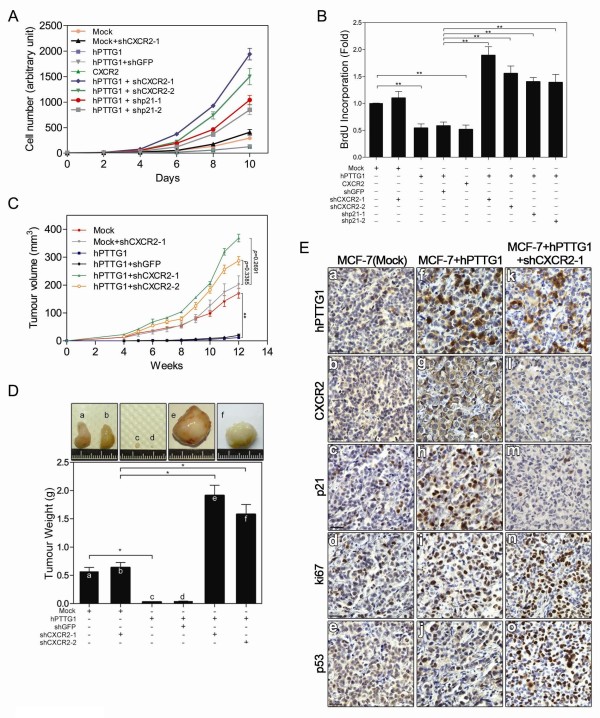
**CXCR2/p21-dependent senescence inhibits hPTTG1-induced tumor growth**. **(A) ***In vitro *growth curves were determined in MCF-7^Mock^cells, MCF-7^hPTTG1 ^cells, MCF-7^CXCR2 ^cells, MCF-7^Mock ^cells infected with shCXCR2-1, and MCF-7^hPTTG1 ^cells infected with shGFP, shCXCR2-1, shCXCR2-2, shp21-1, or shp21-2. **(B) **The colorimetric ELISA assay for BrdU incorporation was performed in representative cells. The results are presented as the mean ± SEM (*n *= 3). **P < 0.01. **(C) **MCF-7^Mock ^cells, MCF-7^Mock ^cells infected with shCXCR2-1, MCF-7^hPTTG1 ^cells, and MCF-7^hPTTG1 ^cells infected with shGFP, shCXCR2-1, or shCXCR2-2 were orthotopically injected into the fourth mammary fat pads of SCID mice. Tumor growth was measured weekly. The experiments were terminated after 12 weeks because of the tumor burden. Representative tumors are shown in **(D)**; *n *= 6 per group per time point. Each time point is presented as the mean ± SEM. **P < 0.01. (D) Tumor weights were measured 12 weeks after orthotopic implantation of representative cells. The representative tumors are shown in the upper panel; *n *= 6 per group. The results are presented as the mean ± SEM. *P < 0.05. (**E**) Representative tumors were embedded in paraffin and sectioned for immunohistologic analysis of representative proteins. Original magnification, ×400. Scale bars, 25 μm.

To confirm whether hPTTG1-induced senescence affects cell proliferation and DNA replication through a CXCR2/p21-dependent mechanism, we carried out cell proliferation and bromodeoxyuridine (BrdU) incorporation assays. In MCF-7 cells, hPTTG1 overexpression significantly inhibited cell proliferation (Figure 5A) and DNA replication (Figure 5B). Conversely, when CXCR2 or p21 was depleted, hPTTG1 overexpression accelerated the proliferation and DNA replication of MCF-7 cells (Figure 5A, B). These results indicate that a CXCR2/p21-dependent mechanism is critical for hPTTG1-induced senescence to inhibit cell proliferation and DNA replication.

To understand the role of the hPTTG1/CXCR2 axis in breast cancer development, we orthotopically transplanted control MCF-7^Mock ^cells, MCF-7^hPTTG1 ^cells, and CXCR2-depleted MCF-7^hPTTG1 ^cells into the fourth mammary fat pad of severe combined immunodeficiency (SCID) mice and monitored tumor growth weekly (Figure 5C). Twelve weeks after transplantation, tumors and lungs were harvested for further investigation. In these mice, hPTTG1 overexpression dramatically inhibited the tumor growth of MCF-7^hPTTG1 ^cells (Figure 5C, Dc). Conversely, when CXCR2 was depleted in MCF-7^hPTTG1 ^cells, hPTTG1 overexpression strongly accelerated the growth of these tumors (Figure 5C, De, f) when compared with CXCR2-knockdown MCF-7^Mock ^cells (Figure 5Db). To confirm the molecular link between hPTTG1 and CXCR2/p21 signaling, we performed an immunohistochemical (IHC) analysis in mouse tumor samples. Compared with control MCF-7^Mock ^tumors (Figure 5Ea, b, c), hPTTG1 overexpression strongly increased the expression of CXCR2 and p21 in MCF-7^hPTTG1 ^tumors (Figure 5Ef, g, h). In these tumors, hPTTG1 overexpression induced strong nuclear localization of p21 (Figure 5Eh), which is a molecular marker of cell-cycle arrest [40]. In addition, knockdown of CXCR2 dramatically limited the expression of p21 in CXCR2-depleted MCF-7^hPTTG1 ^tumors (Figure 5Ek, l, m). Supporting our *in vitro *data, CXCR2 depletion strongly accelerated the proliferation of CXCR2-depleted MCF-7^hPTTG1 ^tumors, which displayed strong ki67 staining, even when p53 was highly induced (Figure 5En, o). Taken together, our findings indicate that CXCR2 plays an important role in inhibiting hPTTG1-induced tumor growth and that it is critical for p21 induction.

**Figure 6:**
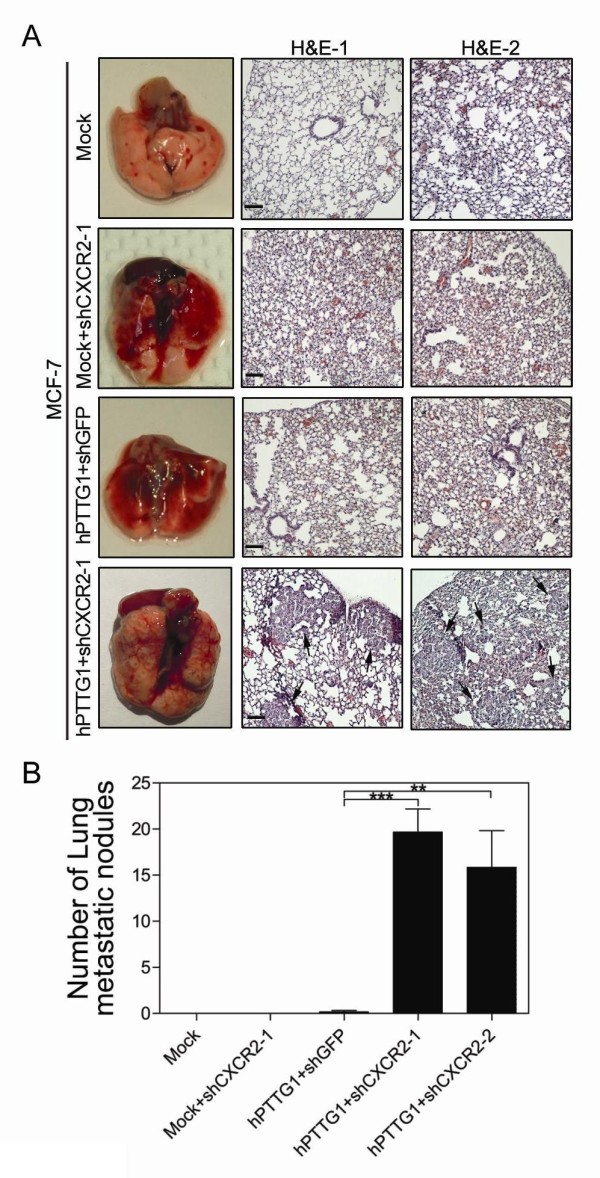
**CXCR2-dependent senescence restrains hPTTG1-induced metastasis**. **(A) **Left column: Images of murine lungs 12 weeks after orthotopic implantation of MCF-7^Mock ^cells, MCF-7^Mock ^cells stably infected with shCXCR2-1, and MCF-7^hPTTG1 ^cells infected with shGFP or shCXCR2-1. Right panel: H&E staining of lungs from mice bearing the indicated tumors; arrows indicate metastatic foci. Magnification, ×200. Scale bars, 100 μm. **(B) **Lung metastases in mice bearing representative tumors were observed by performing H&E analysis of paraffin-embedded lung sections. The numbers of metastatic nodules in the H&E stains were microscopically counted. Quantitative results of the lung metastasis of representative cells (*n *= 6 per group) are presented as the mean ± SEM. **P < 0.01; ***P < 0.0001.

To determine the impact of the hPTTG1/CXCR2 axis on metastasis, we examined lung metastases by performing H&E (hematoxylin and eosin) analysis of lung sections. In these mice, CXCR2-depleted MCF-7^hPTTG1 ^cells metastasized to the lungs in significant numbers 12 weeks after implantation, whereas control MCF-7 cells and MCF-7^hPTTG1 ^cells did not form lung metastatic nodules (Figure 6A, B). Altogether, these results reveal that CXCR2-dependent senescence modulates the role of hPTTG1 overexpression in tumor growth and metastasis. The evasion of CXCR2-dependent senescence (loss of expression of CXCR2 or p21) is important in restoring the oncogenic properties of hPTTG1 overexpression to promote tumor growth and metastasis.

### hPTTG1 affects CXCR2 expression in human invasive ductal carcinomas

**Figure 7:**
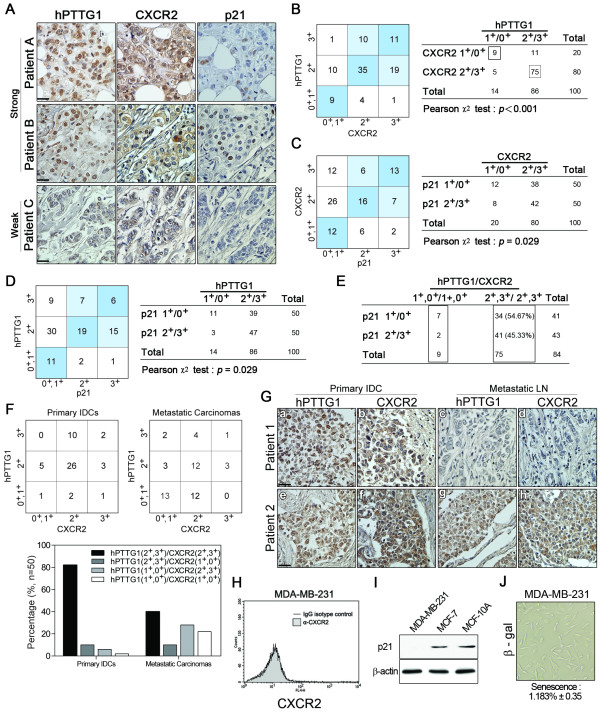
**Positive correlation between hPTTG1, CXCR2, and p21 expression in human breast invasive ductal carcinomas (IDCs)**. **(A) **The expression pattern of hPTTG1, CXCR2, and p21 in representative tumor tissues of patients with breast IDC were immunohistochemically analyzed. Tissues were scored as having strong (3^+^), moderate (2^+^) or weak expression (1^+^/0^+^). The criteria are described in the Materials and methods. Original magnification, ×400. Scale bars, 25 μm. **(B-D) **The expression data for hPTTG1, CXCR2, and p21 in 100 IDC specimens. The correlation between the indicated proteins was analyzed by using the Pearson χ^2 ^test. **(E) **The expression data for p21 in 84 IDC specimens in which hPTTG1 expression was relevant to CXCR2 expression (the 84 specimens are marked with boxes in (B). **(F) **The expression data of hPTTG1 and CXCR2 in 50 primary IDC specimens and 50 matched metastatic carcinomas (upper panel). The specimens were classified into four groups: hPTTG1(2^+^,3^+^)/CXCR2(2^+^,3^+^), hPTTG1(2^+^,3^+^)/CXCR2(1^+^,0^+^), hPTTG1(1^+^,0^+^)/CXCR2(2^+^,3^+^), and hPTTG1(1^+^,0^+^)/CXCR2(1^+^,0^+^). The percentage of hPTTG1/CXCR2-stained specimens of each group is indicated in the lower panel. **(G) **The expression pattern of hPTTG1 and CXCR2 in representative breast IDC and matched metastatic lymph nodes (LNs). Original magnification, ×400. Scale bars, 25 μm. **(H) **MDA-MB-231 cells were stained with FITC-conjugated anti-CXCR2 or isotype control IgG for FACS analysis. **(I) **Cell lysates of MDA-MB-231, MCF-7, and MCF-10A cells were collected for immunoblot analysis. **(J) **MDA-MB-231 cells were plated for senescence assays. The percentage of positively stained cells is presented as the mean ± SEM (*n *= 3).

Invasive ductal carcinoma (IDC) is the most common type of breast cancer [41] and potentially metastasizes to bone, liver, or lung. Therefore, we investigated the clinical relevance of hPTTG1 and CXCR2 expression in human breast IDC specimens. Immunohistochemically stained IDC specimens were scored on the basis of the intensity of hPTTG1 nuclear staining and the percentage of hPTTG1-positive tumor cells [13]. Moreover, the intensity of CXCR2 membrane and cytoplasmic staining in tumor cells was analyzed. In human IDCs (100 cases), hPTTG1 was predominantly expressed in the nuclei (2^+ ^and 3^+^, 86 of 100), whereas CXCR2 staining was present in both the membrane and cytoplasm (2^+ ^and 3^+^, 80 of 100) (Figure 7A, B). Of the 100 specimens evaluated, 75 demonstrated abundant expression of both hPTTG1 and CXCR2, and their expression levels were significantly associated with each other (Figure 7A, Patient A, Patient B; and 7B, *P *< 0.001). Importantly, nine of 14 IDC specimens that were scored as having weak hPTTG1 expression (1^+ ^and 0^+^) also expressed CXCR2 at a very low level (1^+ ^and 0^+^) (Figure 7A, Patient C, and 7B). These results indicate that the expression of hPTTG1 has an important effect on CXCR2 induction in human IDCs.

To support further the molecular link of the hPTTG1/CXCR2/p21 axis that we established *in vitro *and *in vivo*, we analyzed the p21 expression status in these 100 IDC specimens. The expression of p21 was positively correlated with the expression of both CXCR2 (Figure 7C, P = 0.029) and hPTTG1 (Figure 7D, P = 0.029), and these observations suggest that the hPTTG1/CXCR2 axis is important for p21 induction in human IDCs.

In addition, we analyzed the expression status of p21 in 84 IDC specimens in which the hPTTG1 and CXCR2 expression was significantly correlated (Figure 7B, E). Of the 75 specimens that expressed both hPTTG1 and CXCR2 at high levels (hPTTG1: 2^+^/3^+^, CXCR2:2^+^/3^+^), 41 displayed strong (3^+^) or moderate (2^+^) p21 nuclear staining (41 (54.67%) of 75), and 34 (45.33%) demonstrated weak p21 expression (Figure 7E). Of the nine specimens that weakly expressed both hPTTG1 and CXCR2 (hPTTG1, 1^+^/0^+^; CXCR2, 1^+^/0^+^), seven also displayed weak staining of p21 (Figure 7E). These results suggest that p21 mediates hPTTG1/CXCR2-induced senescence. The loss of p21 expression has been observed in several types of cancer [40], and the loss of p21 expression in hPTTG1/CXCR2 double-positive samples (34 (45.33%) of 75) provides a reasonable explanation for how hPTTG1-overexpressing cancer cells manage to escape senescence. Moreover, the tumors that did not lose p21 expression (41 (54.67%) of 75) may carry a mutation in a downstream mediator of hPTTG1/CXCR2/p21 signaling to evade senescence, or the hPTTG1/CXCR2/p21-expressing cells may represent only a portion of a tumor composed of heterogeneous cell types.

### The hPTTG1/CXCR2 axis plays an important role in human breast cancer metastasis

To understand further the role of the hPTTG1/CXCR2 axis in breast cancer metastasis, we collected 50 human primary IDCs and 50 matched lymph nodes with metastatic carcinoma. According to an IHC analysis of hPTTG1 and CXCR2 expression, we classified these primary IDCs and metastatic carcinomas into the following four groups: hPTTG1 (2^+^,3^+^)/CXCR2 (2^+^,3^+^); hPTTG1 (2^+^,3^+^)/CXCR2 (1^+^,0^+^); hPTTG1 (1^+^,0^+^)/CXCR2(2^+^,3^+^); and hPTTG1 (1^+^,0^+^)/CXCR2 (1^+^,0^+^).

Of the primary IDCs, 41 (82%) of 50 expressed both hPTTG1 and CXCR2 at high levels, and only one specimen displayed weak staining of both hPTTG1 and CXCR2 (Figure 7F). This result implies that the hPTTG1/CXCR2 axis plays a metastasis-promoting role in primary tumors.

Interestingly, only 40% of the metastatic carcinomas expressed both hPTTG1 and CXCR2 at high levels (2^+ ^and 3^+^, 20 of 50 in metastatic LNs) (Figure 7F). The number of specimens weakly expressing both hPTTG1 and CXCR2 increased from 2% (one of 50) in primary IDCs to 26% (13 of 50) in metastatic carcinomas (Figure 7F). Notably, eight patients demonstrated abundant expression of both hPTTG1 and CXCR2 in primary IDCs but lost the expression of both hPTTG1 and CXCR2 in metastatic carcinomas (Figure 7Ga, b, c, d). Nineteen patients displayed abundant expression of both hPTTG1 and CXCR2 in both primary and metastatic carcinomas (Figure 7Ge, f, g, h).

Clarifying the complexities of the combinations of hPTTG1-induced senescence, hPTTG1-induced SASP and hPTTG1-induced metastasis in human tumor specimens is difficult. However, these findings have two possible implications. First, considering our findings in the *in vitro *assays (Figure 3C) and human IDCs (Figure 7E), the tumor cells that expressed hPTTG1 and CXCR2 at high levels in both primary and metastatic tumors may escape senescence via the loss of p21 expression. In other words, when the CXCR2/p21-dependent senescence pathway is interrupted (that is, the loss of expression of p21), the evasion of senescence may restore the metastatic potential of hPTTG1 overexpression, as strong staining of hPTTG1 and CXCR2 in metastatic carcinomas was observed (for example, Figure 7G, Patient 2). This conclusion is supported by our previous study [13] demonstrating that hPTTG1 overexpression plays a critical role in promoting the invasion and metastasis of MDA-MB-231 cells that endogenously expressed hPTTG1 at a very high level but expressed CXCR2 (Figure 7H) [42,43] and p21 (Figure 7I) at a very low level and underwent weak senescence (Figure 7J).

Second, when the CXCR2-dependent senescence pathway is intact, it can restrict the growth and metastatic potential of hPTTG1-overexpressing cancer cells. However, given the heterogeneity of breast cancer [41,44], different populations of cancer cells may cooperate to achieve metastasis. Therefore, the hPTTG1-induced SASP may benefit the metastasis of neighboring nonsenescent cancer cells, which would explain why the metastatic nonsenescent cancer cells displayed low expression levels of both hPTTG1 and CXCR2 in metastatic carcinomas (for example, Figure 7G, Patient 1).

### hPTTG1-overexpressing senescent cells promote the invasion and metastasis of neighboring non-senescent cancer cells

**Figure 8:**
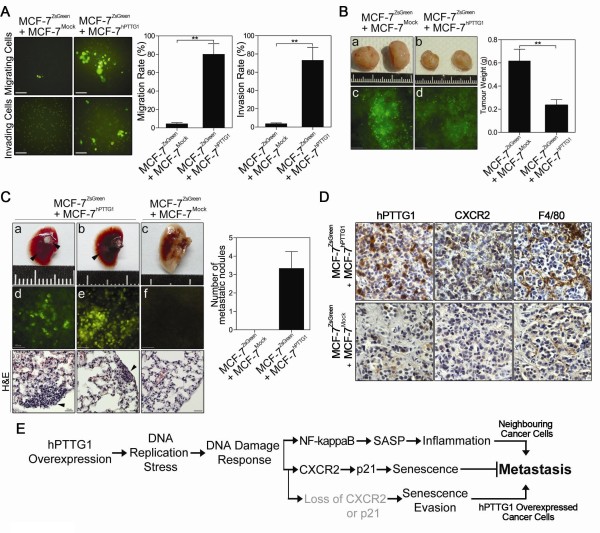
**hPTTG1-induced SASP promotes the metastasis of MCF-7 cells**. **(A) **Migration and invasion assays in the representative cell mixtures. Images show the migrating and invading fluorescent MCF-7 cells (MCF-7^ZsGreen^) in polycarbonate membrane filters (left panel). Original magnification, ×200. Scale bars, 100 μm. Right panels: Quantitative results of the migrating and invading cells. The results are presented as mean ± SEM (*n = 3*). **P < 0.01. **(B) **Tumor weights were measured 14 weeks after orthotopic implantation of representative cell mixtures. The representative tumors are shown in the left upper panel **(a, b)**. The tumor sections were observed with fluorescence microscopy **(c, d)**. Original magnification, ×200. Scale bars, 100 μm. Right panel: Quantitative results of the tumor weight (*n *= 6). The results are presented as the mean ± SEM. **P < 0.001. **(C) **Left upper panel: Images of murine lungs 14 weeks after orthotopic implantation of representative cell mixtures **(a-c)**. Left middle panel: Lung nodules observed with fluorescence microscopy **(d-f)**. Original magnification, ×200. Scale bars, 100 μm. Left lower panel: H&E stain of lungs from mice bearing the indicated tumors; arrows indicate metastatic foci. Original magnification, ×400. Scale bars, 25 μm. Right panel: Quantitative results of the lung metastasis of representative cells (*n *= 6 per group). Data are presented as the mean ± SEM. **(D) **Representative tumors were embedded in paraffin and sectioned for IHC analysis of representative proteins. **(E) **Schematic representation of the role of the hPTTG1/CXCR2 axis in breast cancer development.

To confirm the clinical implications described, we asked whether hPTTG1-overexpressing senescent cells can promote the metastasis of neighboring nonsenescent cells. Senescent cells have been shown to promote the tumor growth and metastasis of neighboring cancer cells by generating a tumor-promoting microenvironment [45-47]. Therefore, we labeled MCF-7 cells with green fluorescent protein (ZsGreen) and mixed these ZsGreen-labeled cells (MCF-7^ZsGreen^, 0.5 × 10^5 ^cells) with MCF-7^Mock ^cells (0.5 × 10^5 ^cells) or MCF-7^hPTTG1 ^cells (0.5 × 10^5 ^cells). By performing cell migration and invasion assays on these two cell mixtures, we found that MCF-7^hPTTG1 ^cells significantly promoted the migration and invasion of MCF-7^ZsGreen ^cells, whereas MCF-7^Mock ^cells did not (Figure 8A).

Furthermore, we orthotopically transplanted the cell mixtures into SCID mice and examined lung metastasis 14 weeks after transplantation. When compared with mice injected with the mixture of MCF-7^ZsGreen ^(2 × 10^5 ^cells) and MCF-7^Mock ^cells (2 × 10^5 ^cells), mice injected with the mixture of MCF-7^ZsGreen ^(2 × 10^5 ^cells) and MCF-7^hPTTG1 ^cells (2 × 10^5 ^cells) displayed a significant reduction in tumor weight (Figure 8Ba, b). Both type of tumors showed heterogeneous green fluorescence, which was exhibited by the MCF-7^ZsGreen ^cells (Figure 8Bc, d). Interestingly, four of the six mice that received transplants with the mixture of MCF-7^ZsGreen ^and MCF-7^hPTTG1 ^cells displayed visible lung metastatic nodules (Figure 8Ca, b). When we directly observed these metastatic nodules with fluorescence microscopy, all visible nodules showed strong green fluorescence (Figure 8Cd, e). Conversely, no observable metastatic nodules or fluorescent micrometastases were found in the lungs of mice given transplants with the mixture of MCF-7^ZsGreen ^and MCF-7^Mock ^cells (Figure 8Cc, f). With H&E analysis of lung sections, the mice injected with the mixture of MCF-7^ZsGreen ^and MCF-7^hPTTG1 ^cells exhibited a significant number of lung metastatic nodules (Figure 8Cg, h), whereas mice injected with MCF-7^ZsGreen ^and MCF-7^Mock ^cells did not exhibit metastatic nodules (Figure 8Ci).

Tumor-associated macrophage infiltration has been demonstrated to promote angiogenesis, invasion, metastasis, and cancer chemoresistance [48,49]. To determine whether hPTTG1-induced SASP provokes an inflammatory response that would promote the metastasis of MCF-7 cells in a mouse model, we examined mouse macrophage infiltration by IHC staining of F4/80, a mouse macrophage-specific marker. Tumors composed of MCF-7^ZsGreen ^and MCF-7^Mock ^cells displayed weak expression of both hPTTG1 and CXCR2 and were mildly infiltrated by mouse macrophages (Figure 8D). Conversely, tumors composed of MCF-7^ZsGreen ^and MCF-7^hPTTG1 ^cells showed strong heterogeneous staining of both hPTTG1 and CXCR2 and demonstrated severe macrophage infiltration (Figure 8D). These results suggest that hPTTG1-induced SASP instigates the inflammatory response and contributes to metastasis.

These data support the implications from the human metastatic carcinomas (Figure 8E). When cells are capable of inducing senescence, CXCR2-dependent senescence can inhibit the proliferation and metastasis of breast cancer cells. However, hPTTG1-induced SASP in the tumor can provoke an inflammatory response and promote the metastasis of nonsenescent neighboring cells. In conclusion, our study elaborates on the role of hPTTG1 in OIS regulation and provides novel evidence by which hPTTG1-overexpressing cells remodel the tumor microenvironment via hPTTG1-induced SASP that promotes breast cancer metastasis.

## Discussion

Our study identifies the role of hPTTG1 overexpression in OIS regulation and breast cancer metastasis (Figure 8E). First, we demonstrated that hPTTG1 overexpression reinforces senescence through a CXCR2/p21-dependent mechanism. Second, CXCR2-dependent senescence is sufficient to restrict the oncogenic potential of hPTTG1 overexpression, and senescence evasion is important for hPTTG1 to exert its oncogenic properties. Third, IHC analyses reveal a positive correlation between hPTTG1 and CXCR2 expression in human IDCs. Finally, we provide novel evidence that the hPTTG1-induced SASP plays an important role in promoting breast cancer metastasis.

*hPTTG1 *is a strong oncogene that promotes the malignant progression of cancers via a variety of mechanisms [6,13,14], and the mechanisms of hPTTG1 accumulation have been investigated by many groups [50-52]. These previous studies emphasized the importance of hPTTG1 overexpression in tumor development, and in support of these studies, both our recent findings [13] and the present study have elucidated the role of hPTTG1 overexpression in breast cancer development and metastasis.

In our previous study, we demonstrated that hPTTG1 overexpression can promote the migration and invasion of MCF-7 cells [13]. However, in the present study, we also demonstrated that hPTTG1 overexpression can reinforce OIS in MCF-7 cells. OIS is a type of premature senescence that does not occur in all cells [20,53,54]. The hPTTG1-overexpressing cells that do not senesce may still retain their capacity for migration and invasion. Moreover, it is possible that hPTTG1/GEF-H1 signaling and hPTTG1/CXCR2 signaling were coactivated in the same cells and that even senescence can limit the proliferation of these cells with higher hPTTG1-induced motilities. Similar contradictory observations were also shown in the first PTTG1 study: PTTG1 overexpression inhibits cell proliferation but promotes cell transformation [1]. Although hPTTG1 overexpression can dramatically limit MCF-7 cell growth, the growth rate of MCF-7^hPTTG1 ^tumors was accelerated for 12 weeks after implantation (Figure 1C). In these mice, lung metastases were observed at approximately 16 to 18 weeks after transplantation, whereas mice with transplanted control MCF-7 cells did not form metastatic nodules in their lungs [13]. Accumulating genetic mutations increase cancer malignancies [55]. We speculate that the accumulating hPTTG1-induced DNA damage provides opportunities for cancer cells to evade senescence. In this situation, senescence would be a selective pressure for senescence-evading cells, and these senescence-evading cells may need more time to grow and metastasize. However, further studies should be conducted to address this issue.

Although several studies have provided convincing evidences that hPTTG1 overexpression plays a tumor-promoting role in various types of cancers [3,12-14,56], the involvement of hPTTG1-induced senescence in cancer development should be carefully considered. For example, the microenvironment constructed by hPTTG1-induced SASP increases the complexity of the role of hPTTG1 in breast cancer tumorigenesis and metastasis. To understand the specific role of hPTTG1-induced senescence in tumorigenesis, we incorporated the concept of intratumoral heterogeneity, which has long been observed in breast cancer [44].

In a tumor composed of heterogeneous populations of cancer cells, hPTTG1-overexpressed senescent cells may benefit the growth and metastasis of neighboring senescence-evading cells. We confirmed this hypothesis by using MCF-7 cells in a coinjection assay in mice. Additionally, MCF-7 cells were originally isolated from metastatic pleural effusions [57], but MCF-7 cells metastasize and invade poorly in mouse models. Given the heterogeneity of breast cancer [41,44], we hypothesised that hPTTG1-overexpressing senescent MCF-7 cells may create a metastasis-promoting microenvironment to restore the metastatic potential of MCF-7 cells, and our results confirmed this hypothesis.

hPTTG1 overexpression has been shown to inhibit cancer cell growth by activating p21 independent of p53 [32]. In this study, we provide evidence that the hPTTG1/CXCR2 axis can induce p21 expression through a p53-independent mechanism. Our findings emphasize the importance of p21 in CXCR2-mediated senescence. CXCR2 has been shown to play critical roles in both tumor promotion and suppression [25,28,58]. Moreover, depletion of CXCR2 has been shown to alleviate the metastatic abilities of melanoma [59,60] and metastatic breast cancer cells [61], but how CXCR2 could play different roles in different cancer cells remains unclear. To explain our finding that p21 knockdown inhibited hPTTG1/CXCR2-induced senescence (Figure 3D, 5A, B), we speculate that p21 is a critical downstream mediator that determines the role of CXCR2 in tumor development. In cells that were capable of inducing p21 expression, CXCR2 induced a senescence response via the activation of p21, whereas in cells in which the p21-dependent mechanism was interrupted, CXCR2 likely exerted its oncogenic properties when senescence was evaded. Senescence is an early event in tumor development and can be induced only in normal cells or low-malignant cancer cells with intact senescence pathways [20]. Highly malignant cancer cells isolated from late-stage tumors or metastatic nodules typically have mutations in senescence pathways that enable the escape from senescence [21,62]. Hence, the loss of p21 or downstream mediators of CXCR2 signaling may transform CXCR2 into a tumor-promoting factor, and this intriguing hypothesis will be further addressed in future studies.

## Conclusions

Overall, our findings provide novel molecular insights into hPTTG1-induced senescence and an understanding of the role of this process in breast cancer metastasis. Thus, the expression status of hPTTG1 should be considered a risk factor for breast cancer metastasis as well as a potential therapeutic target.

## Abbreviations

CXCR2: chemokine (C-X-C motif) receptor 2; DDR: DNA damage response; EGF: epidermal growth factor; ELISA: enzyme-linked immunosorbent assay; GFP: green fluorescent protein; GROα: growth-related oncogene α; H&E: hematoxylin and eosin; HIPAA: Health Insurance Portability and Accountability Act; *hPTTG1*: human pituitary tumor-transforming gene 1; IDC: invasive ductal carcinoma; IHC: immunohistochemistry; IL-8: interleukin-8; LN: lymph node; MIF: migration inhibitory factor; NF-κB: nuclear factor kappa-light-chain-enhancer of activated B cells; OIS: oncogene-induced senescence; qRT-PCR: quantitative reverse transcription polymerase chain reaction; SASP: senescence-associated secretory phenotype; SA-β-Gal: senescence-associated β-galactosidase; shRNA: short-hairpin RNA.

## Competing interests

The authors declare that they have no competing interests.

## Authors' contributions

JWR contributed to the conception and design of the entire study, performed the majority of the experiments, and drafted the manuscript. YCL assisted in cell-culture manipulation and animal studies and edited the manuscripts. IL participated in the luciferase reporter analyses. MHL and CYH contributed to the IHC analyses and the statistical analyses of the human tumor samples. JHC contributed to the study conception and edited the manuscript. All authors read and approved the final manuscript.
